# A Dual Interaction Between the 5′- and 3′-Ends of the Melon Necrotic Spot Virus (MNSV) RNA Genome Is Required for Efficient Cap-Independent Translation

**DOI:** 10.3389/fpls.2018.00625

**Published:** 2018-05-09

**Authors:** Manuel Miras, Ana M. Rodríguez-Hernández, Cristina Romero-López, Alfredo Berzal-Herranz, Jaime Colchero, Miguel A. Aranda, Verónica Truniger

**Affiliations:** ^1^Centro de Edafología y Biología Aplicada del Segura, Consejo Superior de Investigaciones Científicas (CEBAS-CSIC), Murcia, Spain; ^2^Centro de Investigación en Química Aplicada, Consejo Nacional de Ciencia y Tecnología (CONACYT), Saltillo, Mexico; ^3^Instituto de Parasitología y Biomedicina López-Neyra, Consejo Superior de Investigaciones Científicas (IPBLN-CSIC), Granada, Spain; ^4^Departamento de Física, Edificio CIOyN, Universidad de Murcia, Campus de Espinardo, Murcia, Spain

**Keywords:** 3′-CITE, cap-independent translation, MNSV, RNA structure, plant virus, RNA:RNA interactions, translation initiation, translational enhancer

## Abstract

In eukaryotes, the formation of a 5′-cap and 3′-poly(A) dependent protein–protein bridge is required for translation of its mRNAs. In contrast, several plant virus RNA genomes lack both of these mRNA features, but instead have a 3′-CITE (for cap-independent translation enhancer), a RNA element present in their 3′-untranslated region that recruits translation initiation factors and is able to control its cap-independent translation. For several 3′-CITEs, direct RNA-RNA long-distance interactions based on sequence complementarity between the 5′- and 3′-ends are required for efficient translation, as they bring the translation initiation factors bound to the 3′-CITE to the 5′-end. For the carmovirus melon necrotic spot virus (MNSV), a 3′-CITE has been identified, and the presence of its 5′-end *in cis* has been shown to be required for its activity. Here, we analyze the secondary structure of the 5′-end of the MNSV RNA genome and identify two highly conserved nucleotide sequence stretches that are complementary to the apical loop of its 3′-CITE. In *in vivo* cap-independent translation assays with mutant constructs, by disrupting and restoring sequence complementarity, we show that the interaction between the 3′-CITE and at least one complementary sequence in the 5′-end is essential for virus RNA translation, although efficient virus translation and multiplication requires both connections. The complementary sequence stretches are invariant in all MNSV isolates, suggesting that the dual 5′–3′ RNA:RNA interactions are required for optimal MNSV cap-independent translation and multiplication.

## Introduction

Viral mRNAs have evolved numerous mechanisms for recruiting the host’s translational machinery, allowing them to compete with host mRNAs and avoid defense mechanisms that act at the level of translation. Thus, while most plant-encoded mRNAs contain a 5′-cap and a 3′-poly(A) tail that act synergistically to stimulate translation, ∼80% of known positive-strand RNA plant viruses lack one or both of these features in their genomic and subgenomic RNAs (van Regenmortel et al., 2000; Miras et al., 2017a), and they often use their 5′- and/or 3′-termini in alternative gene expression strategies (Nicholson and White, 2011; Truniger et al., 2017). Cap-independent translation in some plant virus RNAs is facilitated by highly structured RNA elements residing within the 5′-untranslated region (5′-UTR), in some cases corresponding to internal ribosomal entry sites (IRES) (Kneller et al., 2006; Zhang et al., 2015; Miras et al., 2017a). In other plant viruses that lack both the cap and 3′-poly(A) tail, such as members of the family *Tombusviridae* and the genus *Luteovirus* (family *Luteoviridae*), RNA elements capable of controlling cap-independent translation residing within their 3′-UTR (abbreviated 3′-CITE for cap-independent translation enhancer) are required for viral RNA translation. Often, *cis*-acting signals residing in the 5′-UTR are also needed for cap-independent translation (Miller and White, 2006; Simon and Miller, 2013; Truniger et al., 2017).

3′-CITEs vary in sequence and folding structure. Based on their RNA structure, seven different types have been identified to date, all in viruses belonging to the family *Tombusviridae*: BTE-like (cloverleaf shape), TED-like (long stem-loop), PTE-like (stem ending with two short connected helical branches), Y-shaped (YSS), I-shaped (ISS), T-shaped (TSS), and CXTE-like 3′-CITEs (Nicholson and White, 2011; Simon and Miller, 2013; Truniger et al., 2017). The last 3′-CITE in this list, CXTE, was identified in two isolates of the carmovirus melon necrotic spot virus (MNSV), MNSV-N and -GX, and was very likely acquired from cucurbit aphid-borne yellows virus (CABYV) through separate interfamilial recombination events (Miras et al., 2014; Truniger et al., 2017). These results show that, in nature, 3′-CITEs are modular and transferable RNA elements. The transfer of 3′-CITEs among viruses confers them with adaptive advantages. 3′-CITEs bind host translation initiation factor eIF4F, as shown for the TED-like (Gazo et al., 2004), YSS (Nicholson et al., 2013), ISS (Nicholson et al., 2010; Miras et al., 2017b), PTE-like (Batten et al., 2006; Wang et al., 2009, 2011) and BTE-like (Treder et al., 2008; Kraft et al., 2013) 3′-CITEs. These results and the observation that several 3′-CITEs continued facilitating cap-independent translation *in vitro* when moved to the 5′-terminus of viral RNAs, thereby replacing their endogenous 5′-UTR (Meulewaeter et al., 1998b; Guo et al., 2000), suggest that the 3′-CITE must be responsible for recruiting the host factors involved in translation initiation and that these must be delivered to the 5′-end near the start codon. Thus, often the presence of both genome ends has been shown to be essential for cap-independent translation (Truniger et al., 2017).

For several types of 3′-CITEs this delivery has been shown or proposed to occur through an interaction based on sequence complementarity between the 3′-CITE and the 5′-end (Simon and Miller, 2013; Truniger et al., 2017). Experimentally, this has been shown for the BTE of barley yellow dwarf virus (BYDV) (Guo et al., 2001), the PTE of saguaro cactus virus (SCV) (Chattopadhyay et al., 2011), the TED of pelargonium line pattern virus (PLPV) (Blanco-Pérez et al., 2016), the YSS of carnation italian ringspot virus (CIRV) (Nicholson and White, 2008; Nicholson et al., 2013) and of tomato bushy stunt virus (TBSV) (Fabian and White, 2004, 2006), and for the ISS of maize necrotic streak virus (MNeSV) (Nicholson et al., 2010). On the other hand, proposed 5′–3′ interactions could not be experimentally confirmed for several viruses, for example, satellite tobacco necrosis virus (STNV) or Red clover necrotic mosaic virus (RCNMV) (Meulewaeter et al., 1998a; Sarawaneeyaruk et al., 2009). Additionally, for tobacco necrosis virus isolate TNV-D, a recent publication shows that the base-pairing between its 5′-UTR and its BTE is not required *in vivo* for efficient virus multiplication (Chkuaseli et al., 2015). 5′–3′ interaction can also occur indirectly through ribosomes as shown for the carmovirus turnip crinkle virus (TCV) (Stupina et al., 2011). For the umbravirus pea enation mosaic virus (PEMV), which contains three 3′-CITEs, direct and indirect modes were proposed to occur (Gao et al., 2012, 2013, 2014).

The carmovirus MNSV, which lacks a 3′-poly(A) tail and a 5′-cap (Díaz et al., 2003, 2004), controls its cap-independent translation with an ISS 3′-CITE named Ma5TE (Miras et al., 2017b). This 3′-CITE, with its 45 nucleotides (nt), is the shortest one known to date and consists of a stem that is closed with an apical 7 nt loop and interrupted by two internal loops that are 3 and 7 nt in length. Translation requires the presence of the 5′-UTR from MNSV *in cis* (Truniger et al., 2008). In addition to the genetic evidence that indicates that MNSV translation is eIF4E-dependent (Nieto et al., 2006; Rodríguez-Hernández et al., 2012), a direct interaction between its Ma5TE and eIF4F has recently been shown (Miras et al., 2017b). Mutations in eIF4E affect its association with eIF4G, reducing Ma5TE activity, thereby showing that both subunits of the eIF4F complex are important. Here we study how this translation initiation complex, which is bound to the Ma5TE, reaches the vicinity of the start codon. We have determined the secondary structure of the 5′-end of the MNSV genome, formed by 5 stem-loops (SL), and identified that the nucleotides from two loops are complementary to the apical loop of the 3′-CITE. Our experimental studies on the cooperation between these sequences show that interaction based on sequence complementarity between at least one of the 5′-end sequences and the 3′-CITE is required for some cap-independent translation of MNSV *in vivo*, but that both 5′–3′ interactions are necessary for efficient translation and thus for wild-type virus multiplication.

## Materials and Methods

### Analysis of RNA Structures

The 84 nt-long 5′-UTR of MNSV, and the 132 nt-long extended sequence of its 5′-end were cloned into a previously described SHAPE cassette plasmid (Wang et al., 2010). This plasmid was linearized with *Sma*I and transcribed using the MEGAshort-script^TM^ Kit (Ambion). Selective 2′-Hydroxyl Acylation analyzed by Primer Extension (SHAPE) experiments using benzoyl cyanide (BzCN) were performed essentially as previously reported (Kraft et al., 2013; Miras et al., 2015). Briefly, 500 ng of RNA refolded in the SHAPE buffer (100 mM KCl, 50 mM HEPES KOH pH7.5, 8 mM MgCl_2_) was treated with 60 mM BzCN (Sigma-Aldrich) for 30 s at 22°C in the absence or presence of Mg^2+^ (0.1, 1, and 4 mM) and resolved on an 8% denaturing polyacrylamide-urea gel after primer extension with a ^32^P-labeled primer complementary to the SHAPE cassette. Normalized BzCN reactivity values for each nucleotide position were calculated by SAFA Footprinting Software (Das et al., 2005). The RNA’s secondary structure was determined with the MC-Fold computer program (Parisien and Major, 2008) using SHAPE reactivity data.

Secondary structure predictions of 5′- and 3′-ends of other viruses from the family *Tombusviridae* were performed with Mfold^1^ or RNAalifold^2^. The prediction of possible RNA interactions including the estimation of their statistical significance was obtained using the Transat web server^3^ (Wiebe and Meyer, 2010). For this, the 5′-end sequence was fused to the 3′-CITE or 3′-UTR sequence, separated by a track of 10 adenosines as described by Diaz-Toledano et al. (2017).

### Reporter Constructs for *in Vivo* Translation Efficiency Assays

5′-UTR deletion mutants were created by PCR amplification (High fidelity system, ROCHE^4^) from the constructs 5′-UTR-luc or 5′-end-luc of MNSV-Mα5 (*luc* = Firefly luciferase gene), followed by directional cloning of the amplified fragment into the *Kpn*I/*Xba*I sites of the luc-3′-UTR-Mα5 plasmid (Truniger et al., 2008). The forward primer started with sequence inside the 5′-UTR, including the T7 promoter sequence and *Kpn*I restriction site, and the reverse primer was complementary to the 3′-end of the *luc* gene followed by *Xba*I [ΔSL 1 (Δ21 nt), ΔSL 1+2 (Δ51 nt) or SL1-3 (Δ73 nt)]. The 3′-UTR deletion mutants were created by PCR amplification from luc-3′-UTR-Mα5 (Truniger et al., 2008), followed by directional cloning into the *Xba*I/*Hpa*I sites of the 5′-UTR-Mα5-luc plasmid. The primer pairs used contained the sequence of the 5′-end of the 3′-UTR preceded by an *Xba*I site (forward) and the reverse sequence inside the 3′-UTR (ΔSL A = Δ43 nt or ΔSL A+B = Δ85 nt of 3′-UTR-Mα5) followed by a *Hpa*I site. The 5′-end-luc-3′-UTR construct was obtained by cloning the first 132 nt of the MNSV-Mα5 genome into the *Kpn*I/*Nco*I sites of the luc-3′-UTR-Mα5 plasmid. In this construct, the ATG of MNSV-ORF1 was in frame with the *luc* gene. Thus, to avoid luciferase synthesis from this start codon, it was mutated by site-directed mutagenesis from ATG to GTG resulting in a 5′-end-luc-3′-UTR. Site-directed mutagenesis on both UTRs and 5′-end was performed by amplification of plasmids 5′-UTR-luc-3′UTR or 5′-end-luc-3′-UTR of MNSV-Mα5 (Truniger et al., 2008) using Pyrobest polymerase^5^ (Takara Bio Inc.) with primers (sense and antisense) containing the desired mutation followed by *Dpn*I digestion to remove the input plasmid [see *in vitro* mutagenesis protocol (Sambrook and Russell, 2001)]. The deletion of Ma5TE^∗^ was obtained following the same method and using PCR amplification primers flanking the deletion. All constructs were verified by sequencing. The *Bsm*I-linearized plasmids were transcribed *in vitro* in the absence or presence of a cap analog (Promega) using the RiboMax^TM^ transcriptase (Promega^6^). 3′-CITE+K (wt and mutants) was added to the 5′-UTR-luc (wt and mutants) by PCR using a primer including the reverse sequence of the end of the *luc* gene plus the 3′-CITE (45 nt) sequence including the sequence added as a clamp, as described by Miras et al. (2017b).

### *In Vivo* Translation Efficiency Assays

*In vivo* translation in melon protoplasts was assayed as described before (Truniger et al., 2008) measuring luciferase activity 4 h after electroporation of the RNA. These experiments were carried out at least four times for each construct. The *in vivo* translation efficiencies of the RNA constructs were analyzed in the presence and absence of the cap (obtained by *in vitro* transcription). The translation efficiencies of the capped RNA constructs were all similar, independently if the UTRs were wild-type or contained mutations. When indicated, hippuristanol (10, 30, 100, 300, and 1000 nM) was added to protoplasts directly after electroporation and kept during incubation. The stability of the assayed RNA constructs in melon protoplasts was assessed by Northern blot analysis of total RNA extracted at 0/2/4 h after electroporation from protoplast samples (after exhaustive washing) using a digoxigenin-labelled luciferase specific cRNA probe. The stability in protoplasts of all the mutant RNA constructs in protoplasts was found to be unchanged as compared to the wild-type constructs, independent of its translation efficiency (not shown).

### Construction and Analysis of MNSV Virus Mutants

The mutations were introduced into the infectious clone pTOPO-MNSV-Mα5 (Diaz et al., 2004). The complete plasmids were PCR-amplified using Pyrobest polymerase (Takara) with primers containing the mutation. They were digested with *Dpn*I to select for the mutant plasmids (Sambrook and Russell, 2001). All constructs were verified by sequencing. The ability of the *in vitro*-transcribed uncapped viral RNAs to multiply in protoplasts of susceptible melon was studied by Northern blot using a digoxigenin-labeled cRNA probe against the 3′-UTR of the MNSV-Mα5 genome (Diaz et al., 2004). Mutations G124C and C125G, located in ORF1, resulted in the change from Ala to Pro and Gly, respectively. But the results obtained allowed for concluding that these amino acid changes did not affect replicase activity, as the mutant viruses with the restored sequence complementarity were able to multiply with similar efficiency as wild-type MNSV-Mα5. The 3′-UTR of the progenies was amplified with MNSV-specific primers by RT-PCR (Roche) and sequenced. The 5′-UTR sequences of the progenies were determined by 5′-RACE using an MNSV-specific primer with sequence complementary to the second ORF and Moloney Murine Leukemia Virus RT (PrimeScript^TM^ Reverse Transcriptase, Takara) followed by PCR amplification (PrimeStar^®^ HS DNA Polymerase, Takara). PCR was performed using the primer used for the reverse transcription and a primer complementary to the first 10 nt of the MNSV genome containing four additional guanosine nucleotides at its 5′-end. The amplified fragment was sequenced. The mutations were stable in the progeny.

## Results

### Analysis of the Secondary Structure of the 5′-UTR of MNSV and Importance of Its Regions in Cap-Independent Translation

**FIGURE 1:**
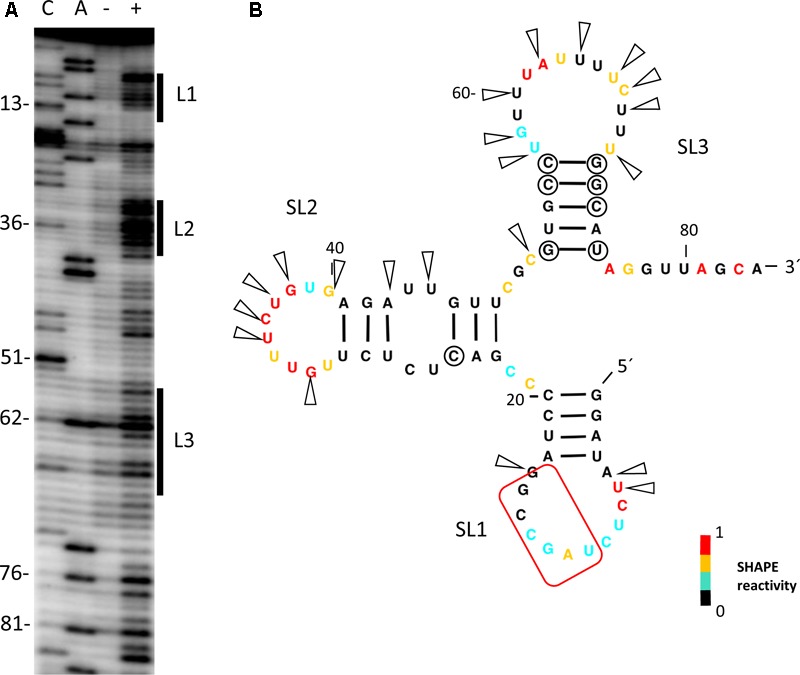
Secondary structure probing of the 5′-UTR of MNSV. **(A)** Structure probing by SHAPE of the 5′-UTR of MNSV-Mα5. Primer extension products separated on denaturing PAGE of RNA treated (+) or untreated (-) with BzCN. The sequencing ladder was generated by reverse transcription of unmodified RNA in the presence of ddGTP (C) or ddTTP (A). Positions of C13, C36, C51, A62, A76, and A81 are indicated on the left. The highly accessible regions forming the loops (L) of stem-loops (SL) 1–3 are marked on the right side of the gel. **(B)** Secondary structure model of probed 5′-UTR. Color-coded bases indicate the levels of BzCN modification, with warmer colors indicating greater modification (inset). Positions of nucleotide variations in the 5′-end sequences of other MNSV isolates that validate this model in double-stranded regions are marked with a circle; these variations do not disrupt base-pairing. Arrows indicate other variable positions in an alignment of MNSV sequences; arrows concentrate in unpaired regions. Nucleotides complementary to the 3′-CITE are marked with a red frame.

Our previous experiments had shown that cap-independent translation of the MNSV isolate MNSV-Mα5 was controlled by its Ma5TE, the translation enhancer present in nearly all the MNSV isolates, and dependent on the presence of the 5′-UTR *in cis* (Truniger et al., 2008; Miras et al., 2017b). Thus, we studied the secondary structure from the 5′-UTR of the MNSV-Mα5 RNA genome by Selective 2′-Hydroxyl Acylation analyzed by Primer Extension (SHAPE) using the chemical benzoyl cyanide (BzCN), which modifies accessible nucleotides in a sequence-independent manner in seconds, forming 2′-O-adducts that block reverse transcriptase (Mortimer and Weeks, 2008). Primer extension revealed three exposed regions (L1–L3) modified by BzCN, corresponding to the loops of the three stem-loop structures (SL) (**Figure 1**). Magnesium titration experiments showed that 5′-UTR folding was independent of this divalent cation (data not shown). Most variations in the 5′-UTR sequences of the MNSV isolates present in GenBank either do not disrupt base-pairing of double-stranded regions (circles in **Figure 1B**), or are preferentially localized in single-stranded regions (arrowheads). This sequence conservation supports the secondary structure model.

**FIGURE 2:**
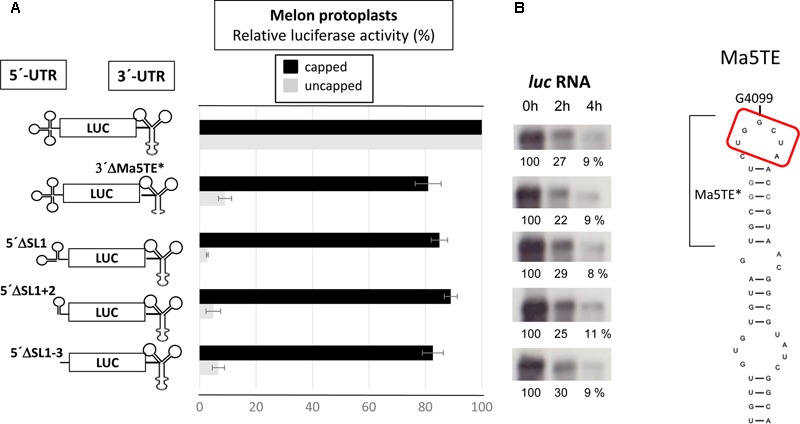
Effect of deletions in the 5′- and 3′-UTRs of MNSV-Mα5 flanking the luciferase reporter gene (*luc*) on translation efficiency. **(A)** Relative luciferase activity corresponding to the *in vivo* translation efficiency in melon protoplasts of the different constructs (horizontal bars; obtained with capped RNA in black and uncapped RNA in gray) corresponding to at least four independent experiments (error bars indicate the standard deviation). The activity of the wild-type construct, 5′-UTR-luc-3′-UTR, was set to 100%. The different constructs are shown at the left: ΔSL1 (Δ21 nt), ΔSL1+2 (Δ51), and ΔSL1+2+3 (Δ73). On the right panel, Northern blot analyses show relative stability of the uncapped RNA constructs in protoplasts 0/2/4 h (h) after electroporation using a luciferase specific RNA probe. (%) = quantification of the detected *luc* RNA relative to the input (0 h) averaged from 3 independent experiments. **(B)** Structure of the Ma5TE, as obtained by structure-probing (Miras et al., 2017b), is shown. The apical SL Ma5TE^∗^ is indicated. Nucleotides complementary to the 5′-end are marked with a red frame.

To identify the regions of the 5′-UTR of MNSV-Mα5 that are important for translation, we studied the effect of deletions of the SL1-3 found in this UTR (total length 84 nt) when flanking the luciferase gene (5′-UTR-luc-3′-UTR) on cap-independent translation in melon protoplasts (Truniger et al., 2008). The results obtained in the *in vivo* translation experiments of melon protoplasts showed that deletion of the first 21 nt corresponding to SL1 and thus, both other deletions [SL1+2 (Δ51) and SL1+2+3 (Δ73)] as well, strongly affected the cap-independent translation efficiency of the constructs (**Figure 2A**), suggesting that these first nucleotides were critical for Ma5TE activity. Also the apical small SL of the Ma5TE (**Figure 2B**), defined previously (Miras et al., 2017b), was shown to be critical for its activity.

### Importance of Sequence Complementarity Between Both UTRs of MNSV-Mα5 for the *in Vivo* Translation Efficiency of *luc*-Constructs

**FIGURE 3:**
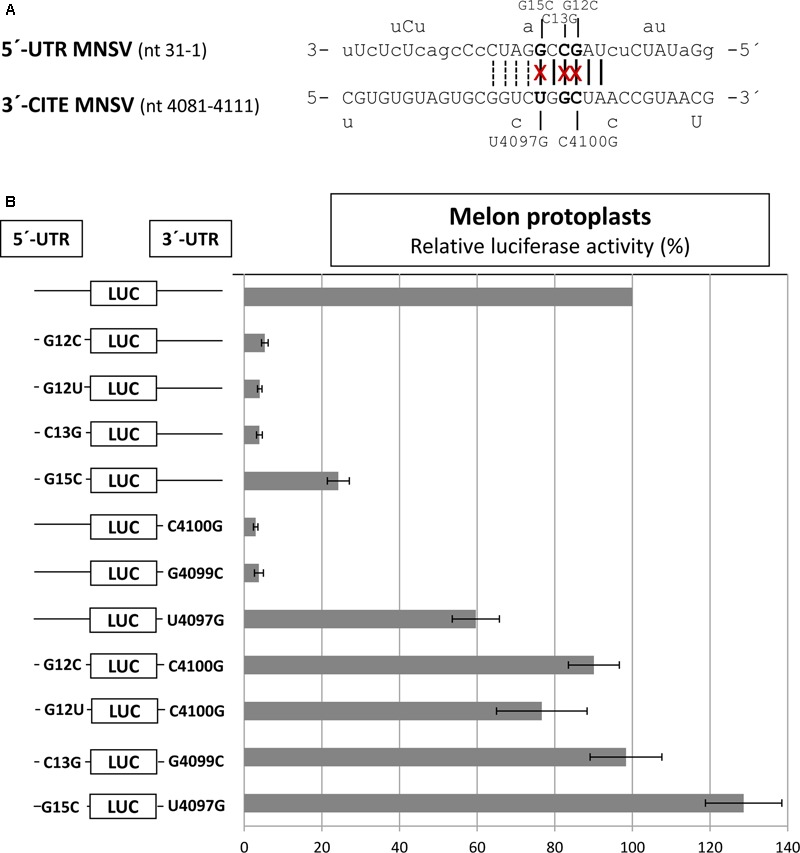
Importance of sequence complementarity between both UTRs of MNSV-Mα5 for the *in vivo* translation efficiency of *luc*-constructs. **(A)** Sequences of the first 31 nucleotides of the 5′-UTR of MNSV-Mα5 in 3′–5′-sense and of a Ma5TE fragment including its apical loop in 5′–3′ sense. Nucleotides of the 5′-UTR complementary to the 3′-CITE appear in uppercase letters, while at the top and below the nucleotide variations found in other MNSVs (GenBank) are shown (Truniger et al., 2008) (**Supplementary Figures S1A,B**). Sequence complementarity to the 3′-CITE conserved in all MNSV 5′-UTRs is marked with vertical lines, additional complementarity in MNSV-Mα5 by dotted lines. The nucleotides mutated here are marked with an “x.” **(B)** Horizontal bars show *in vivo* translation efficiencies measured as luciferase activities obtained in melon protoplasts of mutant RNA constructs relative to that of the wild-type construct, 5′-UTR-luc-3′-UTR (set to 100%). Error bars represent the standard deviation of at least four independent experiments.

By comparing the sequences of the first 21 nt of MNSV with that of its 3′-CITE, six complementary nucleotides that were invariant in the genomes of the MNSV isolates available in GenBank (**Supplementary Figure S1**), could be identified. These complementary nucleotides were present in loops in the secondary structures assayed, in SL1 of the 5′-UTR (**Figure 1B**) and in the apical loop of the 3′-CITE (**Figure 2A**). Complementarity could be extended from 6 to 10 nucleotides for MNSV-Mα5 (**Figure 3A**). To study the possibility of a direct 5′–3′ interaction based on nucleotide complementarity, we exchanged single nucleotides from the loop of SL1 (G12C, G12U, C13G, and G15C) and the corresponding complementary nucleotides from the 3′-CITE (C4100G, G4099C, and U4097G) in the 5′-UTR-luc-3′-UTR construct (Truniger et al., 2008) (**Figure 3A**). We studied the effects of these mutations on the *in vivo* cap-independent translation efficiency. These analyses showed that each of the single point mutations in the 5′- or 3′-UTR caused a strong reduction in the translation efficiency of the construct, resulting in less than 10% of the luciferase activity obtained with the wild-type construct (**Figure 3B**). Mutations G15C and U4097G caused smaller reductions, approximately 30 and 60% of the luciferase activity obtained with the wild-type construct, respectively. Importantly, the introduction of the complementary mutations (G12C/C4100G, G12U/C4100G, C13G/G4099C, and G15C/U4097G) restored the translational activity to levels similar to those shown by the wild-type construct. The fact that both G12C and G12U mutations were able to compensate C4100G, confirmed that sequence complementarity between both UTRs was important for cap-independent translation controlled by the Ma5TE. Additionally, the fact that the translation efficiency of the G12U/C4100G construct was lower than that of the wild-type and the G12C/C4100G constructs, suggests that the weaker U–G interaction leads to reduced translation. In line with this result, the stronger C–G interaction of the G15C/U4097G construct resulted in a higher translation efficiency than the wild-type construct.

In agreement with this notion, for the ISS of MNeSV, it has been shown that an interaction based on sequence complementarity between the nucleotides of the apical loop of the ISS and the first loop in the predicted CIRV 5′-UTR structure was required for 3′-CITE activity (Nicholson et al., 2010).

### Prediction of Additional RNA Interactions Between the Ends of MNSV Genomes

**FIGURE 4:**
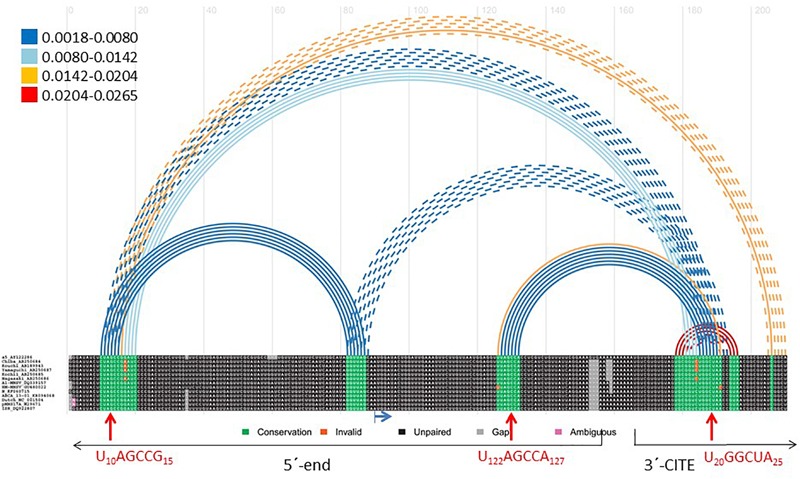
Transat prediction of RNA interactions between MNSV genome ends. Bioinformatic prediction of possible functional interactions between the ends of MNSV genomes (Transat) (Wiebe and Meyer, 2010). Aligned sequences of 13 MNSV isolates available in GenBank (accession numbers in **Supplementary Figure S1**) including the first 150 nt of the genome and the Ma5TE sequence (45 nt), separated by 10 adenosines (as described for the identification of interactions by Diaz-Toledano et al. (2017). Color of arched lines connecting interacting base pairs correspond to the estimated statistical significance of the interactions (*P*-value, color code shown at the left). Broken lines depict mutually exclusive helices. The maximal *P*-value threshold for the prediction was set at 0.03, the minimal stem length was 6. In the alignment, highly conserved sequences appear in green. The blue horizontal arrow marks the start codon of ORF1. Red vertical arrows mark the complementary nucleotides (amplified below) predicted to interact.

**FIGURE 5:**
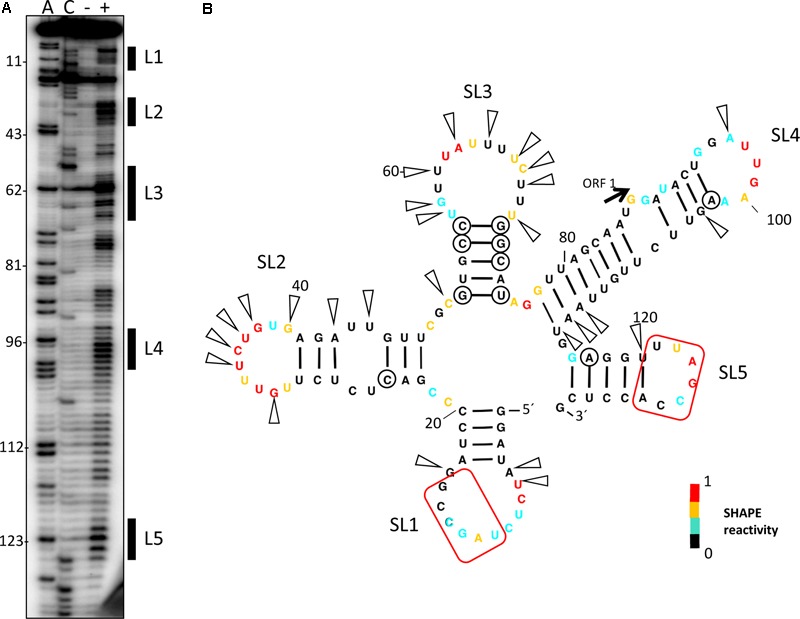
Secondary structure probing of the 5′-end of the MNSV genome. **(A)** Structure probing by SHAPE of the 5′-end of MNSV-Mα5 (132 nt). Primer extension products separated on denaturing PAGE of RNA treated (+) or untreated (–) with BzCN. The sequencing ladder was generated by reverse transcription of unmodified RNA in the presence of dideoxy-ATP (ddTTP; A) or ddGTP (C). Positions of some A’s, starting at A11, are indicated on the left. The highly accessible regions forming the loops (L) of stem-loops (SL) 1–5 are marked on the right side of the gel. **(B)** Secondary structure model of probed 5′-end. Color-coded bases indicate the levels of BzCN modification, with warmer colors indicating greater modification (inset). The start codon of ORF1 is indicated. Positions of nucleotide variations in the 5′-end sequences of other MNSV isolates that validate this model in double-stranded regions are marked with a circle; these variations do not disrupt base-pairing. Arrows indicate other variable positions in an alignment of MNSV sequences (sequence alignment see **Supplementary Figure S1**); arrows concentrate in unpaired regions. Nucleotides complementary to the 3′-CITE are marked with a red frame.

We used the Transat bioinformatics tool, that detects conserved helices of high statistical significance, including pseudo-knotted, transient and alternative structures starting with a multiple sequence alignment (Wiebe and Meyer, 2010), to predict interactions between the ends of MNSV genomes. Here, we used an alignment of the 5′-ends (including the first 150 nt) and the nearly invariant 3′-CITEs (45 nt) of the MNSV genomes (see **Supplementary Figure S1**). The program predicted the interaction between the apical loop of the Ma5TE (U20–A25) and the six nucleotides of the 5′-UTR (U10–G15) that we had identified to be important for translation (**Figure 4**). Additionally, the tool also predicted an interaction between the same nucleotides of the Ma5TE and the six nucleotides located in ORF1, U122–A127 (red arrows). Both 5′–3′ interactions were predicted with high statistical significance (**Figure 4**), also when the analysis was performed with the complete 3′-UTR (**Supplementary Figure S2**). Previously, it had been suggested that the sequence present in ORF1 could possibly play a role in 5′–3′ interaction (Simon, 2015). Transat also predicted another dual interaction with high statistical significance (**Figure 4**), between G77–G82 and C9–C14 or C4100–C4106, but our assayed 5′-end (**Figure 5**) and Ma5TE (**Figure 2B**) RNA structures did not support this prediction, as part of the nucleotides involved were present in stems. But the possibility that these predicted interactions are transient and dynamic cannot be excluded.

To study the possible importance of the predicted interaction between nucleotides located in ORF1, U122–A127, and the Ma5TE, we first examined the secondary structure of the 5′-end of the MNSV-Mα5 RNA genome with SHAPE (**Figure 5**). The first three SLs coincided with the UTR structure (**Figure 1**). The ORF1 sequence (from nt 121–127), complementary to the Ma5TE, was located in the SL5 loop. Thus, as it was unpaired, it may be involved in an interaction, based on sequence complementarity. Additionally, the alignment of the 5′-end and 3′-CITE sequences of the MNSV genomes present in GenBank showed that the complementary sequence stretches in the UTRs and in ORF1 were invariant in all MNSV isolates (**Supplementary Figure S1**) (Truniger et al., 2008; Miras et al., 2017b), supporting their importance. Thus, interaction of the 3′-CITE with the 5′-UTR seemed to be a general mechanism in MNSV translation, but an additional interaction with ORF1 could still exist.

### Importance of the Complementary Sequence Stretch in ORF1 in Ma5TE-Controlled Cap-Independent Translation

**FIGURE 6:**
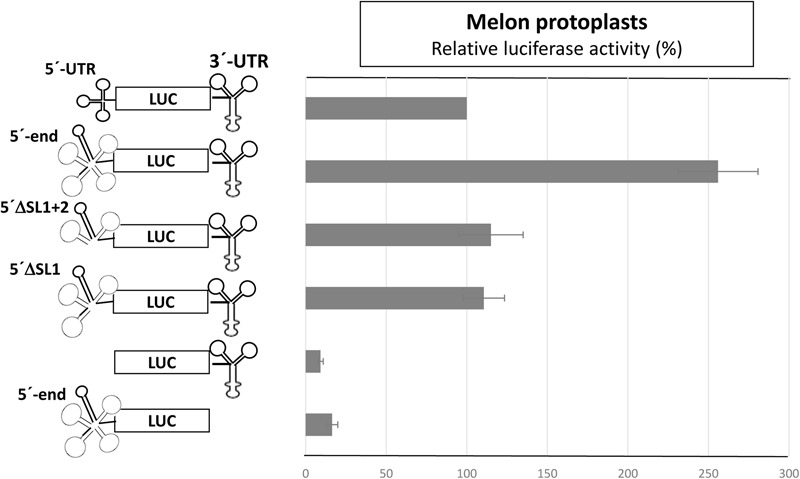
Involvement of a sequence in ORF1 in cap-independent translation. *In vivo* cap-independent translation assays in melon protoplasts of constructs of the luciferase gene flanked by the 3′-UTR and either the 5′-UTR (84 nt) or the 5′-end (132 nt) of the MNSV genome and subsequent deletions: SL1 (Δ21 nt) or SL1+2 (Δ51 nt). The activity of the wild-type construct, 5′-UTR-luc-3′-UTR, was set to 100%. Error bars represent the standard deviation of at least four independent experiments. The different constructs are shown at the left.

To analyze whether the complementarity between Ma5TE and both 5′-end sequence stretches was important for Ma5TE-mediated cap-independent translation, we added part of ORF1 (48 nt) to the 5′-UTR of MNSV-Mα5, flanking the *luc* gene (5′end-luc-3′-UTR). Since the AUG from MNSV-ORF1 was in frame with the *luc* gene and to avoid luciferase synthesis from this start codon, it was mutated by site-directed mutagenesis from AUG to GUG, resulting in the 5′-end-luc-3′-UTR. In this work we differentiate between the genomic 5′-UTR (untranslated sequence) and the 5′-end, including additionally sequence downstream from the start codon. The cap-independent translation activity in melon protoplasts of the previous 5′-UTR-luc-3′-UTR construct was 2.5-fold lower than that of the new one (**Figure 6**, first and second bars), suggesting that the added sequence played a role in translation. Additionally, the deletion of SL1 or SL1+2 of the 5′end-luc-3′-UTR constructs reduced the translation efficiencies only to levels similar to that of the UTR-construct (**Figure 6**, third and fourth bars) and not less than 10% as shown in **Figure 2**. Thus, the added sequence seemed to be able to compensate for the loss of the 5′-UTR-3′-CITE interaction of the deletion constructs, suggesting that it could contain a second 3′-CITE-interacting sequence that is important for efficient *in vivo* cap-independent translation.

**FIGURE 7:**
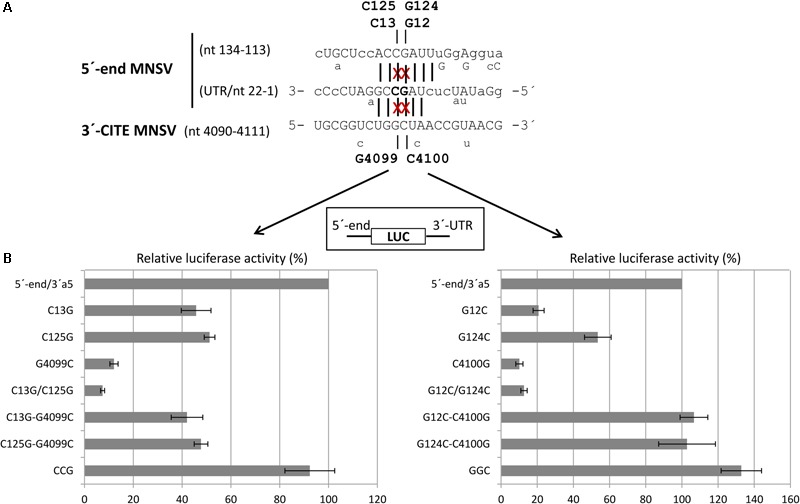
Importance of sequence complementarity between the Ma5TE and two regions in the 5′-end of MNSV-Mα5 for the *in vivo* translation efficiency of luc-constructs. **(A)** Sequence in the 3′–5′-sense of the first 22 nucleotides of the 5′-UTR and nucleotides 134–113 of ORF1, partially complementary to the sequence in the loop of Ma5TE shown below in 5′–3′ sense. Sequence variations present in the 5′-end and 3′-CITE of other sequenced MNSV isolates is indicated below the sequences (alignment **Supplementary Figure S1**). Sequence complementarity with the 3′-CITE is shown with vertical lines (conserved in all MNSVs) and capital letters (in MNSV-Mα5). The positions of the complementary point mutations introduced in the 5′- end and 3′-UTR of MNSV-Mα5 luc constructs (G12C, G124C, C13G, C125G, G4099C, and C4100G) are marked with “x”. **(B)** Horizontal bars represent luciferase activities relative to the wild-type construct (100%) corresponding to *in vivo* cap-independent translation efficiencies obtained in melon protoplasts. Error bars represent the standard deviation of at least four independent experiments. GGC and CCG correspond to triple complementary mutants: GGC-G12C/G124C/C4100G and CCG-C13G/C125G/G4099C.

Thus, we studied the effect of point mutations in the 5′-end nucleotides complementary to the same Ma5TE sequence using the 5′end-luc-3′-UTR construct, disrupting one or both possible complementary interactions (**Figure 7A**). The luciferase activities obtained *in vivo* with these wild-type and mutant RNAs (**Figure 7B**) revealed that single point mutations in one of the two sequence stretches of the 5′-end (C13G, C125G, G12C, and G124C) reduced the translation efficiency to 20–50% of the wild-type activity, while reduction was much stronger for the constructs with the single mutations in the 3′-CITE or the double mutations in the 5′-end (approximately 10% of the wild-type activity; G4099C, C4100G, C13G/C125G, and G12C/G124C). In these constructs, both possible complementary 5′–3′ interactions were disrupted, explaining the low translation efficiency. In agreement with this view, restoring the nucleotide complementarity between the mutated Ma5TE and one of the two 5′-end sequences led to a partial recovery of the translation efficiency (C13G-G4099C, C125G-G4099C), and only the restoring of the nucleotide complementarity with both 5′-end sequences (CCG = C13G, C125G- plus G4099C) led to its complete recovery. The results obtained with the constructs, including mutation C4100G [G12C-C4100G, G124C-C4100G and GGC (=G12C, G124C plus C4100G)] also support this explanation, although the translation percentages with respect to the wild-type construct were higher, maybe due to the change of C4100 into G was of some advantage for Ma5TE activity or because G–G mismatches have been shown to be more stable than the C–C mismatches (Kierzek et al., 1999). These results suggest that both complementary sequences in the 5′-end are necessary for highly efficient Ma5TE-mediated cap-independent translation *in vivo*, while complementarity with at least one of the 5′-end sequences is required for Ma5TE activity. The mutational analysis was repeated with 5′end-luc-3′CITE constructs (**Supplementary Figure S3**), with the 3′-UTR exchanged in just the Ma5TE. Similar results were obtained, confirming that this dual interaction was only Ma5TE-dependent and independent from the rest of the 3′-UTR.

### Importance of Sequence Complementarity Between Both Ends of the MNSV-Mα5 Genome for Virus Multiplication

**FIGURE 8:**
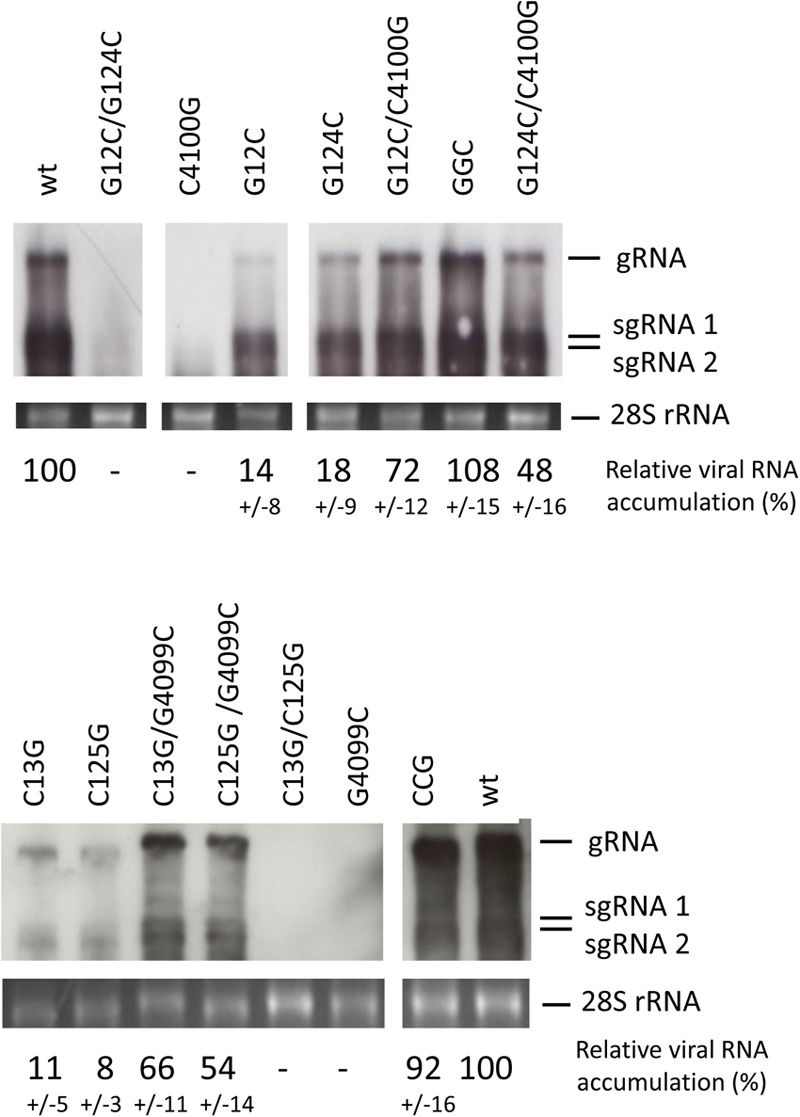
Importance of sequence complementarity between both genomic 5′-end and 3′-UTRs for the multiplication of MNSV-Mα5. Multiplication of MNSV-Mα5 mutants in melon protoplasts as detected by Northern blot analysis. Positions of genomic (gRNA) and subgenomic RNAs (sgRNA) are indicated. GGC and CCG are triple mutants with restored complementarity: GGC – G12C/G124C/C4100G and CCG – C13G/C125G/G4099C. The cRNA probe was complementary to the 3′-UTR of MNSV-Mα5. The amount of total RNA loaded was visualized by methylene blue staining of the 28S rRNA (bottom panel). Percentage of viral RNA accumulation in relation to wild-type MNSV-Mα5 (wt) ± standard deviation is indicated (average from three independent experiments).

The results obtained above with the reporter constructs and the high conservation of this dual sequence complementarity in the genomes of different MNSV isolates (**Supplementary Figure S1**) suggested that these interactions could be important for virus multiplication. Thus, we studied the effect of partial or total loss of the sequence complementarities identified above on the MNSV-Mα5 genome multiplication capacity in melon protoplasts. As shown in the Northern blots in **Figure 8**, mutant viruses with both complementary interactions disrupted (with a single point mutation in the Ma5TE sequence or with mutations in both complementary 5-end sequence stretches) were unable to multiply in melon protoplasts (G12C/G124C, C4100G, C13G/C125G, and G4099C), correlating with the negative effect of these mutations on the translation efficiency observed in the previous experiments. On the other hand, disruption of only one of the complementary sequence interactions by single mutations on the 5′-end allowed some virus multiplication (G12C, G124C, C13G, and C125G). In the presence of the corresponding complementary mutation in the Ma5TE, virus multiplication was higher (G12C/C4100G, G124C/C4100G, C13G/G4099C, and C125G/G4099C), but only when sequence complementarity between both sequence stretches was restored (GGC, CCG) did virus multiplication reach wild-type levels. Thus, in agreement with the previous results, both complementary interactions seemed to be required for wild-type multiplication efficiency.

### Additional Factors That Are Important for Cap-Independent Translation Controlled by the Mα5TE

**FIGURE 9:**
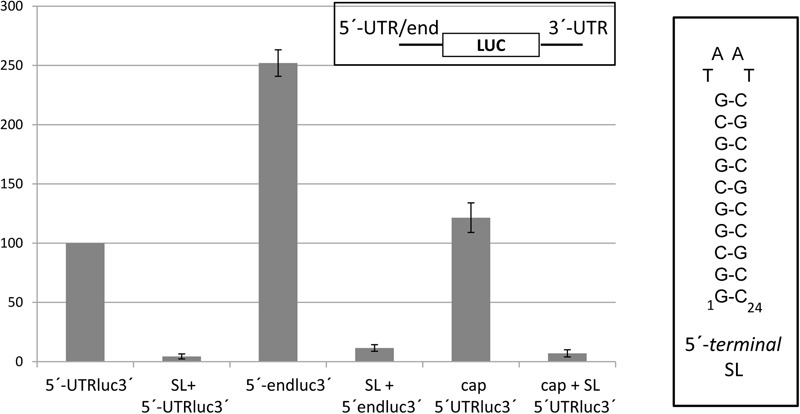
Effect of insertion of a SL-structure at the 5′-terminus of reporter constructs on Ma5TE-controlled cap-independent translation. A stable SL-structure (Δ*G* = –27.30 kcal/mol, 10 bp helix; shown at the right) was introduced to the 5′-terminus of the reporter constructs of the luciferase gene flanked by the 5′-UTR/end and 3′-UTR of MNSV-Mα5. The translation efficiency in melon protoplasts of the different constructs referred to that of the wild-type 5′-UTR-luc-3′-UTR, set as 100%. Cap-dependent translation (cap, cap+SL) of the luciferase gene flanked by the plasmid sequence (Truniger et al., 2008). Error bars represent the standard deviation of at least four independent experiments.

Ribosome scanning occurs from the 5′-end of the viral genome in several cases of 3′-CITE-mediated translation, as shown for BTE (Guo et al., 2001). In that case, addition of a sequence stretch that folds into a stable SL at the 5′-end has been shown to avoid ribosome loading, inhibiting BTE-mediated translation. To study if during Ma5TE-mediated translation the ribosome also scans from the 5′-end, we added a similar stable SL to the 5′-terminus of the luc constructs flanked by the 5′-end or 5′-UTR and the 3′-UTR of MNSV-Mα5. Translation assays *in vivo* in melon protoplasts (**Figure 9**) showed that the addition of this stable SL to the RNA constructs inhibited not only cap-dependent translation (last two columns), but also cap-independent translation mediated by Ma5TE, independently of the length of the 5′-end (columns 1–4). This result suggests that efficient cap-independent translation controlled by the Ma5TE requires ribosome scanning from the 5′-end.

**FIGURE 10:**
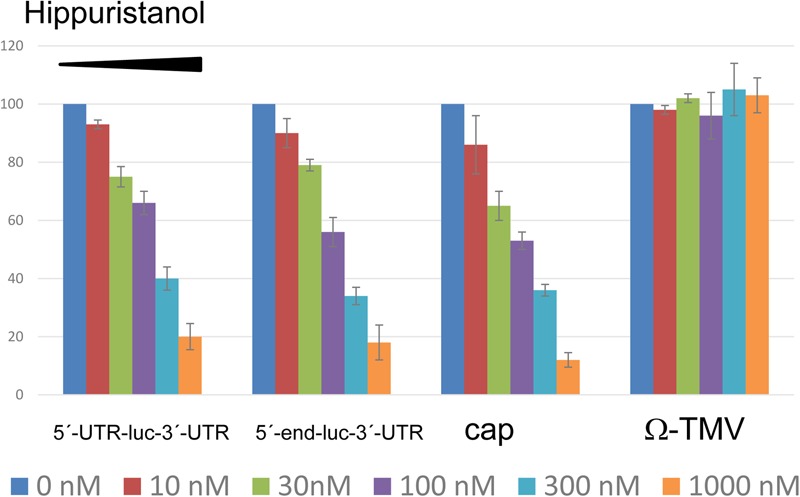
Inhibition of translation by hippuristanol. Translation efficiency of different luciferase constructs in the presence of increasing concentrations of hippuristanol relative to the activity in its absence. The first two constructs show Ma5TE-mediated translation in the presence of 5′-UTR or the 5′-end of the MNSV genome. Controls: cap-dependent translation of luciferase gene flanked by the plasmid sequence (Truniger et al., 2008) and uncapped eIF4A-independent (Sakharov and Agalarov, 2016) translation controlled by the Ω-element of TMV (Ω-luc-polyA60), kindly obtained from A. Miller. This last construct contains nucleotides 1–68 of the TMV genomic RNA, including the omega sequence (Fan et al., 2012). Capped construct consisted of the luciferase gene flanked by sequence corresponding to plasmid pGL3 of 38 nt at the 5′-end and 83 nt at the 3′-end, as described previously (Truniger et al., 2008).

Not much is known about the requirement of eIF4A in cap-independent 3′-CITE-mediated translation. Thus, we wanted to learn if eIF4A could be involved in Ma5TE-mediated translation. For this, translation assays in the presence of hippuristanol (kindly obtained from J. Pelletier), an eIF4A-inhibitor, were performed (Cencic and Pelletier, 2016), with this compound added to melon protoplasts after transfection with luc-constructs. This compound inhibits eIF4A activity in plants (wheat) as well (Roberts et al., 2017). Our results showed (**Figure 10**) that the addition of increasing concentrations of hippuristanol to melon protoplasts inhibited Ma5TE-mediated translation, similar to cap-dependent translation. But translation mediated by the W-element of TMV, known to be independent of eIF4A (Sakharov and Agalarov, 2016), was not affected. These results suggest that eIF4A could be involved in Ma5TE-mediated cap-independent translation.

## Discussion

Most genomic RNAs 3′-CITEs from viruses belonging to the family *Tombusviridae* have been shown or proposed to interact with the 5′-end by sequence complementarity, with this interaction being important for cap-independent translation of the virus genome (Simon and Miller, 2013). By compensatory mutational analysis resulting in disruption and restoration of base-pairing, we have shown that efficient translation and multiplication of the carmovirus MNSV requires that its Ma5TE interact with two sequence stretches at its 5′-end. The maintenance of at least one of these interactions is essential for translation and multiplication. These complementary sequence stretches are invariant in all MNSV isolates, suggesting that these dual interactions between both genome ends are a general mechanism required for cap-independent translation and multiplication of MNSVs.

To learn if these dual 5′–3′ interactions could be predicted to be a more general mechanism of carmoviruses or viruses with ISS, we analyzed their 5′-end and 3′-CITE sequences. For these viruses, 5′-end interactions based on sequence complementarity with their 3′-CITEs have been shown or proposed to reside either in the vicinity of the first SL of the predicted 5′-UTR structure or within the first ORF (Simon and Miller, 2013). We could identify in all cases of viruses with ISS and some carmoviruses sequence stretches containing five or more nucleotides complementary to the 3′-CITE loop in both the 5′-UTR and ORF1 (**Supplementary Tables S1, S2**). The nucleotides involved were mostly unpaired in the predictions (with Mfold) of the 5′-end secondary structure of these virus genomes, and could therefore possibly be involved in an interaction with its 3′-CITE (**Supplementary Figure S4**). Out of these viruses, only for carnation mottle virus (CarMV), enough sequenced isolates exist in GenBank for performing the bioinformatic analysis for predicting RNA interactions using Transat. Interestingly, also for this virus a dual interaction could be predicted (**Supplementary Figure S5**). Thus, such dual interactions may also exist in other viruses of the family *Tombusviridae* (containing different 3′-CITEs). But to draw any conclusions, this interaction should first be studied in detail in each virus.

With regards to the 5′–3′ interactions based on sequence complementarity proposed for other viruses with 3′-CITEs, some of the published experimental results could be explained with this dual interaction: for example, for the YSS of the tombusvirus CIRV, a 5′-UTR-3′-CITE interaction has been shown to exist, but mutations in the complementary sequence of the YSS reduced virus multiplication much more than mutations in the 5′-UTR, and restoring complementarity in the 5′-UTR mutant did not result in increased virus multiplication (Nicholson and White, 2008). The authors explain these results with the higher stability of the G–G versus the C–C mismatch, but the presence of a second 5′–3′ interaction required for efficient translation activity, as described here for MNSV, could also provide an additional explanation. Also for this tombusvirus, a second complementary 6 nt sequence stretch located in ORF1 (nt 152–157) was identified, apart from the one found in the 5′-UTR (nt 18–22). In the Mfold RNA structure prediction of the CIRV 5′-end these complementary sequence stretches were unpaired (**Supplementary Figure S4C**) and, thus, they may interact with a complementary sequence. Also, in some other cases, the finding that single mutations in the 5′-end of the predicted 5′–3′-interacting sequence stretches only had a slight negative effect on virus translation, while the introduction of the complementary mutation in the opposite end did not restore translation to wild-type levels, may indicate that dual interactions are involved in efficient cap-independent translation (Meulewaeter et al., 1998a,b; Guo et al., 2001; Sarawaneeyaruk et al., 2009). On the other hand, experimental results obtained for SCV (Chattopadhyay et al., 2011), TBSV (Fabian and White, 2004, 2006) and MNeSV (Nicholson et al., 2010) did not support such a dual interaction. The results for MNeSV were obtained with a chimeric CIRV virus, with its YSS exchanged with the ISS from MNeSV; thus, the identified 5′–3′ interaction occured between the ISS of MNeSV and the 5′-UTR of CIRV (Nicholson et al., 2010). In conclusion, to know if a dual interaction between both genome ends is a more general mechanism required in cap-independent translation, further studies are needed.

We have recently shown that the 3′-CITE of MNSV binds eIF4F through eIF4E (Miras et al., 2017b). The 5′–3′ interactions identified in the present work could be responsible for bringing the translation initiation complex bound to the Ma5TE to the 5′-end of the genomic RNA. Since both 5′-end sequences are complementary to the same sequence of the 3′-CITE, these interactions should be mutually exclusive, and may occur one after the other. While the interaction of the Ma5TE with the two sequences of the 5′-end is required for efficient translation and virus multiplication, some translation still occurs if one of these interactions is missing and is only abolished if both interactions fail. Thus, although it is advantageous for virus RNA translation to have both connections, they are not essential. The translation efficiency could be increased when both connections are present, as their cooperative binding could help to keep the 3′-CITE in close proximity of the 5′-end. Thus, if one 5′- interaction is disrupted by the scanning or translating activity of the ribosome, the other could be binding the just freed 3′-CITE again. Additionally, we have observed that the secondary structure predictions of the 5′-UTR and the 5′-end of MNSV using RNAalifold (structural alignment using sequences from MNSV isolates available in GenBank) and Mfold differed from our probed structures (**Supplementary Figure S6** and **Figures 1B, 5**). The difference was in the first 22 nucleotides, which form SL1 in our probed structure. Thus, while in our probed structure the nucleotides interacting with the 3′-CITE were in the SL1 loop, in the structure prediction they appeared paired to the complementary sequence either at the 5′-UTR end or at the beginning of ORF1. If both conformations have a role and coexist in the genome, the dual interaction would be advantageous.

An example of two eIF4E-binding structures in the 5′-end of a mRNA has been described for the human histone H4-mRNA (Martin et al., 2011), which has a 5′-cap and also an eIF4E-sensitivity element (4E-SE) in the coding region. The cap-structure is hidden inside a secondary structure, which first has to be melted by eIF4A to become accessible to eIF4E. Thus, the authors proposed that this helicase would be released to the 5′-end by the interaction of eIF4F through eIF4E with the 4E-SE, which would result in the melting of the secondary structure, freeing the 5′-cap. A mutant H4-mRNA with 4 nt changes in the 4E-SE showed a twofold lower translation efficiency than the wild-type H4-mRNA. A similar mechanism could be proposed for translation of the MNSV genome: although the interaction of the 3′-CITE with the ORF1 sequence is sufficient for translation, eIF4A could be released through this interaction and could melt the upstream secondary structure. In favor of this proposal, our results suggest that MNSV Ma5TE-driven translation is eIF4A-dependent, as previously shown only for another 3′-CITE, the one found in BYDV (Zhao et al., 2017). Also, our results suggest that the ribosome must be loaded at the 5′-terminus of the MNSV RNA, as previously shown for BYDV (Rakotondrafara et al., 2006), TBSV (Fabian and White, 2006) and for the chimeric CIRV/MNeSV virus (Nicholson et al., 2010). Dynamic RNA structures that play different roles have been recently described in other viral genomes (Kuhlmann et al., 2016; Liu et al., 2016; Romero-López and Berzal-Herranz, 2017).

It is often difficult to show 5′–3′ RNA:RNA interactions experimentally: if they are transient or require proteins, these interactions may be difficult to detect biochemically. Also, if the interacting nucleotides have additional functions, no compensation will be observed by complementary mutations. Our results of the *in vivo* compensatory mutational analysis clearly support the interactions between the 5′-end and 3′-CITE of MNSV-Mα5 based on sequence complementarity. But we were not able to show this interaction *in vitro*, neither with RNA transcripts of different lengths using gel retardation protocols that were successful for other RNA–RNA interactions (Fabian and White, 2006; Romero-López and Berzal-Herranz, 2009; Nicholson et al., 2010), nor performing atomic force microscopy (AFM) studies (Alvarez et al., 2005) to visualize circularization with the translationally active luciferase construct (data not shown). Thus, we propose that in this case, additional protein factors, possibly the translation initiation factors bound to the 3′-CITE, may stabilize this 5′–3′ interaction. Also, in other cases it has been proposed that host protein(s) enhance base-pairing (Rakotondrafara et al., 2006), as found for the Norwalk virus (Sandoval-Jaime and Gutiérrez-Escolano, 2009). The *in vitro* formation of a tripartite complex of 5′-UTR-3′-CITE-eIF4F, which is required for efficient ribosome recruitment to the start codon, has been shown for MNeSV (Nicholson et al., 2010).

## Conclusion

We show that at least one interaction based on sequence complementarity between the Ma5TE and the 5′-end of the MNSV RNA genome is essential for virus translation and multiplication, but a second interaction is advantageous for these viral functions.

## Author Contributions

MM, AR-H, JC, and VT performed the experiments. MM, CR-L, JC, and VT analyzed the data. MA, MM, and VT conceived the study. MM, MA, CR-L, AB-H, and VT wrote the manuscript. All authors read and approved the final manuscript.

## Conflict of Interest Statement

The authors declare that the research was conducted in the absence of any commercial or financial relationships that could be construed as a potential conflict of interest.

## Footnotes

1 http://unafold.rna.albany.edu/?q=mfold/RNA-Folding-Form

2 http://rna.tbi.univie.ac.at/cgi-bin/RNAWebSuite/RNAalifold.cgi

3 http://www.e-rna.org/transat/

4 http://www.roche.com/index.htm

5 http://www.takara-bio.com/

6 http://www.promega.com/

## Supplementary Material

FIGURE S1 Nucleotide sequence conservation of the 5′-end and 3′-CITE of MNSV genomes. **(A)** Sequence alignment of the 5′-end sequences of MNSV isolates using ClustalW. **(B)**. Sequence alignment of the 3′-CITE sequences of MNSV isolates using ClustalW. The GenBank accession numbers of MNSV sequences included in the alignments are Mα5 (MNSV-Mα5)-AY122286, Ma71-EU589619, Ma24-EU589616, Pa58-EU589620, Chiba-AB250684, Kouchi-AB189943, Yamaguchi-AB250687, Kochi-AB250685, Nagasaki-AB250686, AI-DQ339157, HM-GU480022, ABCA-KR094068, N-KF060715, ISR-DQ922807, Dutch-NC001504, 264-AY330700, and 17A/01A-M29671. The nucleotides conserved in all the sequences are marked with an asterisk (^∗^) below the aligned sequences. The complementary sequence stretches involved in the two 5′–3′ interactions, as well as the ORF1 start codon, are boxed.

FIGURE S2 Prediction of RNA interactions between the 5′-end and 3′-UTR of MNSV genomes. Bioinformatic prediction of possible functional interactions between the ends of MNSV genomes (Transat) (Wiebe and Meyer, 2010). Aligned sequences of 13 MNSV genomes (GenBank accession numbers in **Supplementary Figure S1**) including the first 150 nt and the 3′-UTR sequence, separated by 10 adenosines. Color of arched lines connecting interacting base pairs correspond to the estimated statistical significance of the interactions (*P*-value, color code at the left). Broken line arcs depict mutually exclusive helices. The maximal *P*-value threshold for the prediction was set at 0.02, the minimal stem length was 6. Highly conserved sequences appear in green in the alignment. The blue horizontal arrow marks the start codon of ORF1. Red vertical arrows mark the complementary nucleotides (amplified below) predicted to interact.

FIGURE S3 Importance of sequence complementarity for translation controlled by Ma5TE. *In vivo* cap-independent translation efficiency obtained in melon protoplasts of constructs with only the Ma5TE (45 nt) instead of the 3′-UTR flanking the 3′-end of the luciferase gene. A structure-stabilizing G-C clamp (+K) as described by Miras et al. (2017b) was added to the Ma5TE, as shown on the left. On the right: relative luciferase activity (%) shown as horizontal bars for each construct, as indicated. The activity of the wild-type construct 5′-end-luc-Ma5TE+K was set as 100%. Error bars represent the standard deviation of at least four independent experiments.

FIGURE S4 Viral 5′-end secondary structure models. Schematic representation of the secondary structure prediction obtained with Mfold of the 5′-ends (between 180 and 240 nt) of the viral genomes **(A)** with I-shaped 3′-CITEs; **(B)** of the other carmoviruses apart from MNSV with proposed or identified 3′-CITEs; **(C)** of the tombusvirus CIRV. The arrows indicate the location of the identified complementary sequence stretches. Triangles denote the start of ORF1.

FIGURE S5 Prediction of RNA interactions between the 5′-end and 3′-UTR of CarMV genomes. Bioinformatic prediction of possible functional interactions between the ends of MNSV genomes (Transat). Aligned sequences of all known CarMV genomes available in GenBank including the first 150 nt and the 3′-UTR sequence, separated by 10 adenosines. Color of arched lines connecting interacting bases correspond to the estimated statistical significance of the interactions (*P*-value). Broken line arcs depict mutually exclusive helices. The maximal *P*-value threshold for the prediction was set at 0.03, the minimal stem length was 6. Highly conserved sequences appear in green in the alignment. The blue horizontal arrow marks the start codon of ORF1. Red vertical arrows mark the complementary nucleotides (amplified below) predicted to interact.

FIGURE S6 Secondary structure prediction of the 5′-end of the MNSV genome. Prediction of the secondary structure using RNAalifold generated by structural alignment of the **(A)** 5′-UTRs and **(B)** 5′-ends (150 nt) of the MNSV genome sequences available in GenBank. Structure drawing with conservation annotation. Positions of nucleotide variations in the 5′-end sequences of other MNSV isolates that validate this model in double-stranded regions are marked with a circle; these variations do not disrupt base-pairing. Arrows indicate start codon (blue) and 3′-CITE interacting sequences (red).

TABLE S1 Sequence stretches of the 5′-UTR or ORF1 with complementarity to I-shaped 3′-CITEs.

TABLE S2 Sequence stretches localized in the 5′-UTR or ORF1 of carmovirus genomes with complementarity to their 3′-CITEs.
