# Supplementary figures and images for: A Dual Interaction Between the 5′- and 3′-Ends of the Melon Necrotic Spot Virus (MNSV) RNA Genome Is Required for Efficient Cap-Independent Translation
Source: Front Plant Sci. 2018 May 9;9:625. doi: 10.3389/fpls.2018.00625 (PMC5954562; doi:10.3389/fpls.2018.00625)

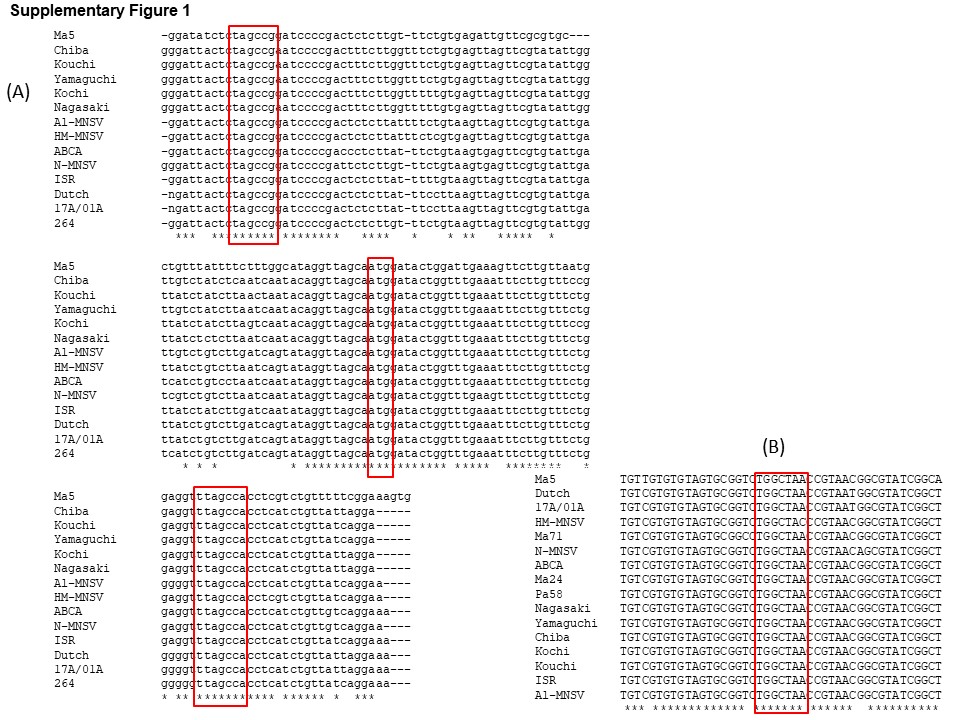

Supplement: FIGURE S1 — Nucleotide sequence conservation of the 5′-end and 3′-CITE of MNSV genomes. (A) Sequence alignment of the 5′-end sequences of MNSV isolates using ClustalW. (B). Sequence alignment of the 3′-CITE sequences of MNSV isolates using ClustalW. The GenBank accession numbers of MNSV sequences included in the alignments are Mα5 (MNSV-Mα5)-AY122286, Ma71-EU589619, Ma24-EU589616, Pa58-EU589620, Chiba-AB250684, Kouchi-AB189943, Yamaguchi-AB250687, Kochi-AB250685, Nagasaki-AB250686, AI-DQ339157, HM-GU480022, ABCA-KR094068, N-KF060715, ISR-DQ922807, Dutch-NC001504, 264-AY330700, and 17A/01A-M29671. The nucleotides conserved in all the sequences are marked with an asterisk (∗) below the aligned sequences. The complementary sequence stretches involved in the two 5′–3′ interactions, as well as the ORF1 start codon, are boxed. [file Image_1.JPEG]

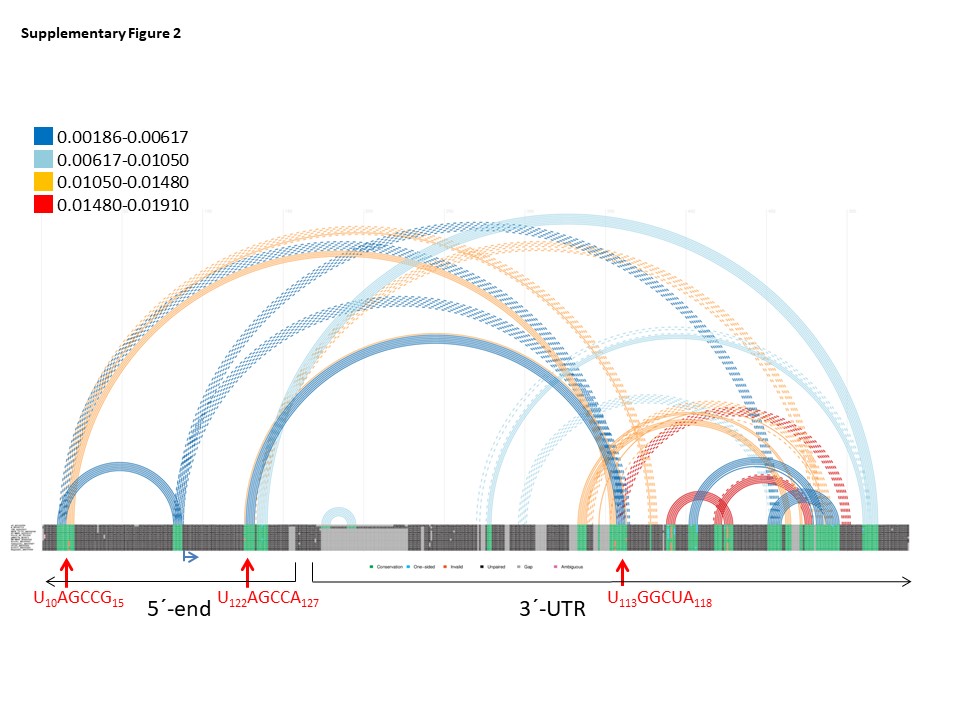

Supplement: FIGURE S2 — Prediction of RNA interactions between the 5′-end and 3′-UTR of MNSV genomes. Bioinformatic prediction of possible functional interactions between the ends of MNSV genomes (Transat) (Wiebe and Meyer, 2010). Aligned sequences of 13 MNSV genomes (GenBank accession numbers in Supplementary Figure S1) including the first 150 nt and the 3′-UTR sequence, separated by 10 adenosines. Color of arched lines connecting interacting base pairs correspond to the estimated statistical significance of the interactions (P-value, color code at the left). Broken line arcs depict mutually exclusive helices. The maximal P-value threshold for the prediction was set at 0.02, the minimal stem length was 6. Highly conserved sequences appear in green in the alignment. The blue horizontal arrow marks the start codon of ORF1. Red vertical arrows mark the complementary nucleotides (amplified below) predicted to interact. [file Image_2.JPEG]

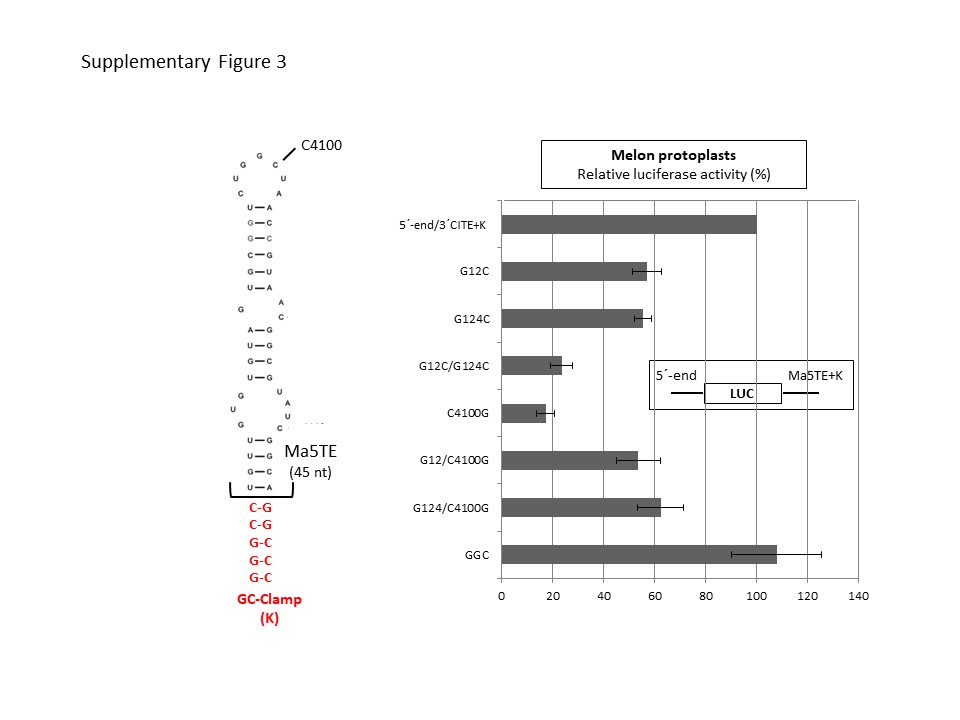

Supplement: FIGURE S3 — Importance of sequence complementarity for translation controlled by Ma5TE. In vivo cap-independent translation efficiency obtained in melon protoplasts of constructs with only the Ma5TE (45 nt) instead of the 3′-UTR flanking the 3′-end of the luciferase gene. A structure-stabilizing G-C clamp (+K) as described by Miras et al. (2017b) was added to the Ma5TE, as shown on the left. On the right: relative luciferase activity (%) shown as horizontal bars for each construct, as indicated. The activity of the wild-type construct 5′-end-luc-Ma5TE+K was set as 100%. Error bars represent the standard deviation of at least four independent experiments. [file Image_3.JPEG]

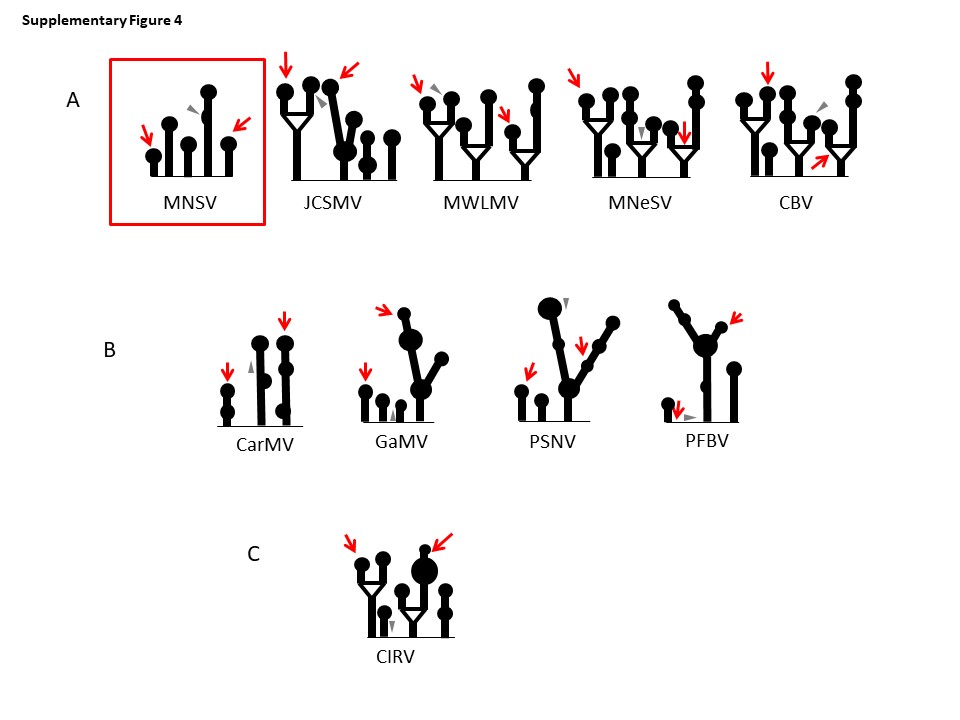

Supplement: FIGURE S4 — Viral 5′-end secondary structure models. Schematic representation of the secondary structure prediction obtained with Mfold of the 5′-ends (between 180 and 240 nt) of the viral genomes (A) with I-shaped 3′-CITEs; (B) of the other carmoviruses apart from MNSV with proposed or identified 3′-CITEs; (C) of the tombusvirus CIRV. The arrows indicate the location of the identified complementary sequence stretches. Triangles denote the start of ORF1. [file Image_4.JPEG]

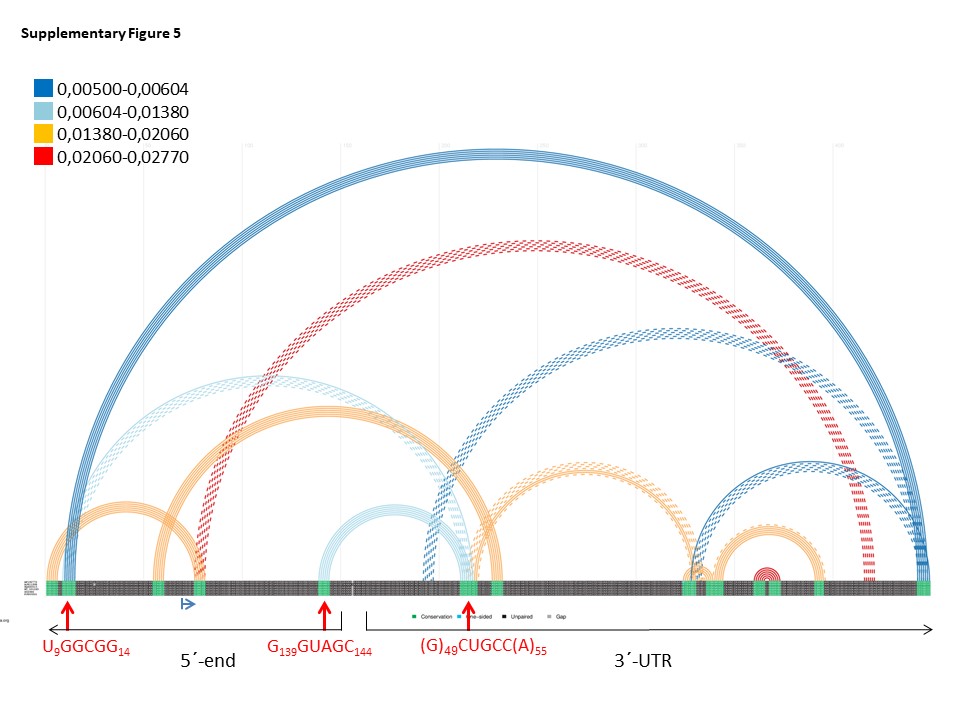

Supplement: FIGURE S5 — Prediction of RNA interactions between the 5′-end and 3′-UTR of CarMV genomes. Bioinformatic prediction of possible functional interactions between the ends of MNSV genomes (Transat). Aligned sequences of all known CarMV genomes available in GenBank including the first 150 nt and the 3′-UTR sequence, separated by 10 adenosines. Color of arched lines connecting interacting bases correspond to the estimated statistical significance of the interactions (P-value). Broken line arcs depict mutually exclusive helices. The maximal P-value threshold for the prediction was set at 0.03, the minimal stem length was 6. Highly conserved sequences appear in green in the alignment. The blue horizontal arrow marks the start codon of ORF1. Red vertical arrows mark the complementary nucleotides (amplified below) predicted to interact. [file Image_5.JPEG]

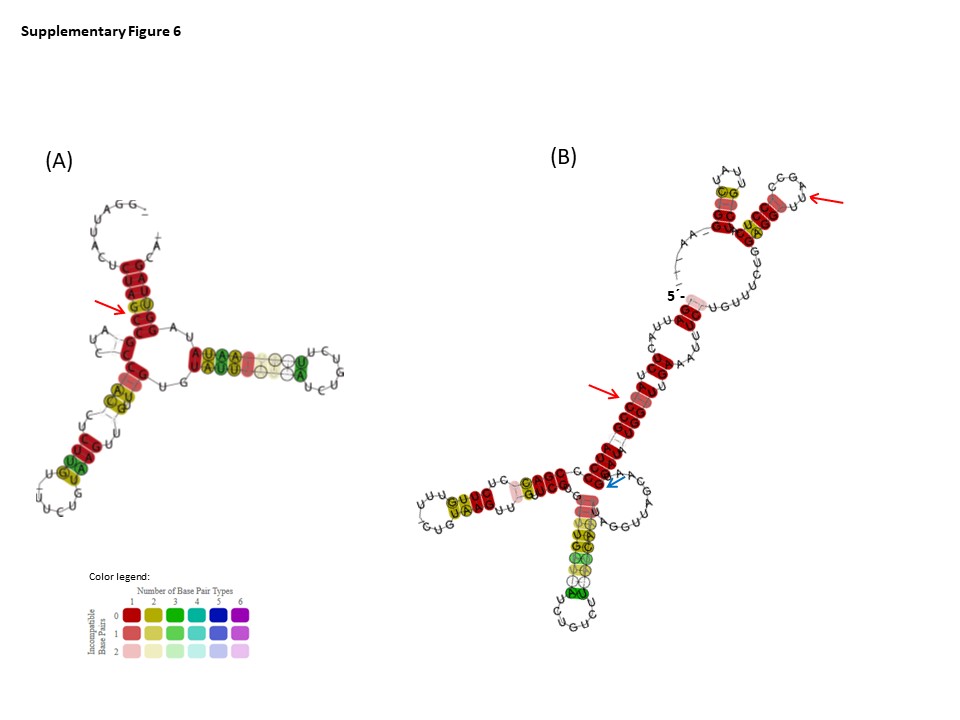

Supplement: FIGURE S6 — Secondary structure prediction of the 5′-end of the MNSV genome. Prediction of the secondary structure using RNAalifold generated by structural alignment of the (A) 5′-UTRs and (B) 5′-ends (150 nt) of the MNSV genome sequences available in GenBank. Structure drawing with conservation annotation. Positions of nucleotide variations in the 5′-end sequences of other MNSV isolates that validate this model in double-stranded regions are marked with a circle; these variations do not disrupt base-pairing. Arrows indicate start codon (blue) and 3′-CITE interacting sequences (red). [file Image_6.JPEG]
