# Supplementary material for: A Dual Interaction Between the 5′- and 3′-Ends of the Melon Necrotic Spot Virus (MNSV) RNA Genome Is Required for Efficient Cap-Independent Translation
Source: Front Plant Sci. 2018 May 9;9:625. doi: 10.3389/fpls.2018.00625 (PMC5954562; doi:10.3389/fpls.2018.00625)
Supplement: TABLE S1 — Sequence stretches of the 5′-UTR or ORF1 with complementarity to I-shaped 3′-CITEs. [file Table_1.docx]

**Supplementary Table 1:**

Sequence stretches of the 5´-UTR or ORF1 with complementarity to I-shaped 3´-CITEs.

| **ISS** | **5´-UTR** | **Genus** | | **5´-UTR** | | **5´-ORF** | **interaction** |
| --- | --- | --- | --- | --- | --- | --- | --- |
| **MNSV** | 84 nt | Carmovirus | UAGCCG (10) (+) AUCGGU | | UUAGCCA (121)(+)  AAUCGGU | | both |
| **MNeSV** | 122 nt | Tombusvirus | UGACCAG (16) (+)  ACUGGUC | | GGUGAUU (171)(+)  CCACUGG | | UTR of CIRV |
| **MWLMV** | 40 nt | Aureusvirus | AACCA (19) (+)  UUGGU | | GACCGU (122)(+)  UUGGUG | | ? |
| **JCSMV** | 45 nt | Aureusvirus | UUGACCA (8) (+)  AACUGGU | | UUGGUU (100)(+)  AACUGG | | ? |
| **CBV** | 124 nt | Tombusvirus | GUUGU (17) (+)  UGGUG | | GAACCA (154)(+)  CUUGGU | | ? |

The length of the 5´-UTR and the virus genus are indicated (second and third column). Nucleotides of the 5´-UTR (column 4) or the ORF1 (column 5) complementary to the 3´-CITE (lower line) are shown. The number in brackets indicates the location of the first complementary nucleotide in the 5´end sequence (upper line). The (+) indicates that these nucleotides are unpaired in the structure prediction by Mfold. The last column indicates that besides this study with MNSV, only for the MNeSV ISS, a 5´-3´ interaction with the 5´-UTR of CIRV has been shown to exist ([Nicholson et al., 2010](#_ENREF_36)). Cucumber bulgarian virus (CBV), maize white line mosaic virus (MWLMV), johnsongrass chlorotic stripe mosaic virus (JCSMV).
