# Supplementary material for: A Dual Interaction Between the 5′- and 3′-Ends of the Melon Necrotic Spot Virus (MNSV) RNA Genome Is Required for Efficient Cap-Independent Translation
Source: Front Plant Sci. 2018 May 9;9:625. doi: 10.3389/fpls.2018.00625 (PMC5954562; doi:10.3389/fpls.2018.00625)
Supplement: TABLE S2 — Sequence stretches localized in the 5′-UTR or ORF1 of carmovirus genomes with complementarity to their 3′-CITEs. [file Table_2.docx]

**Supplementary Table 2:**

Sequence stretches localized in the 5´-UTR or ORF1 of carmovirus genomes with complementarity to their 3´-CITEs.

| **Carmovirus** | **5´-UTR** | **3´-CITE** | **5´-UTR** | **5´-ORF** | **interaction** | |
| --- | --- | --- | --- | --- | --- | --- |
| **MNSV** | 84 nt | ISS | UAGCCG (10) (+)  AUCGGU | UUAGCCA (121) (+)  AAUCGGU | | both |
| **PSNV** | 134 nt | PTE | GUUGG (14) (+)  CGGUU | GCCGG (171) (+/-)  CGGUU | | UTR |
| **CarMV** | 70 nt | PTE | UGGCGG (9) (+)  ACCGUC | GGUAGC (139) (+)  CCGUCG | | UTR |
| **GaMV** | 42 nt | PTE | CGCCAA (9) (+)  GCGGUU | GUUGG (96) (+)  CGGUU | | UTR |
| **PFBV** | 32 nt | PTE | UCUGG (21) (+)  AGACC | gCUGGCAGG (99)(+)  aGACCGUCU | | ORF |

The length of the 5´-UTR and the shape of the 3´-CITE are indicated (second and third column). Only carmoviruses with proposed/determined 3´-CITEs are shown, excluding TCV, for which 5´-3´ interaction has been shown to occur directly through ribosomes ([Stupina et al., 2011](#_ENREF_52)) and viruses of the genus *Pelarspovirus* ([Scheets et al., 2015](#_ENREF_49)), previously classified as carmoviruses. Nucleotides of the 5´-UTR (column 4) or the ORF1 (column 5) complementary to the 3´-CITE (lower line) are shown. The number in brackets indicates the location of the first complementary nucleotide in the 5´end sequence (upper line). The (+) or (+/-) indicate the possibility of these nucleotides being unpaired, based on the structure prediction by Mfold. The last column indicates which of these two sequence stretches has been predicted ([Simon and Miller, 2013](#_ENREF_51)) or shown (for SCV ([Chattopadhyay et al., 2011](#_ENREF_5))) to be involved in 5´-3´ interaction. Pea stem necrosis virus (PSNV); Carnation mottle virus (CarMV); galinsoga mosaic virus (GaMV); pelargonium flower break virus (PFBV).
